# Normalization of the percentage of signal recovery derived from dynamic susceptibility contrast perfusion MRI in brain tumors

**DOI:** 10.1007/s00234-025-03580-7

**Published:** 2025-03-11

**Authors:** Francesco Sanvito, Jingwen Yao, Nicholas S. Cho, Donatello Telesca, Noriko Salamon, Timothy F. Cloughesy, Benjamin M. Ellingson

**Affiliations:** 1 UCLA Brain Tumor Imaging Laboratory (BTIL), Center for Computer Vision and Imaging Biomarkers, University of California Los Angeles, Los Angeles, CA USA; 2 Department of Radiological Sciences, David Geffen School of Medicine, University of California Los Angeles, Los Angeles, CA USA; 3 Department of Bioengineering, Henry Samueli School of Engineering and Applied Science, University of California Los Angeles, Los Angeles, CA USA; 4 Medical Scientist Training Program, David Geffen School of Medicine, University of California, Los Angeles, Los Angeles, CA USA; 5 Department of Biostatistics, University of California, Los Angeles, USA; 6 Department of Neurology, David Geffen School of Medicine, University of California Los Angeles, Los Angeles, CA USA; 7 Department of Neurosurgery, David Geffen School of Medicine, University of California, Los Angeles, Los Angeles, CA USA; 8 Department of Psychiatry and Biobehavioral Sciences, David Geffen School of Medicine, University of California, Los Angeles, Los Angeles, CA USA

**Keywords:** Perfusion imaging, Percentage of signal recovery, Normalization, Brain tumors, Glioblastoma

## Abstract

The universalizability of the metric percentage of signal recovery (PSR) derived from dynamic susceptibility contrast (DSC) perfusion MRI is limited by its dependency of acquisition parameters. In this technical assessment, we tested different reference tissues for PSR normalization and found the normal-appearing white matter (NAWM) to have the least inter-patient variability when using a fixed PSR-optimized protocol. A logarithmic normalization using NAWM improved the consistency of PSR values when a cohort of brain tumor patients was analyzed by synthetically changing acquisition parameters (while keeping the protocol *uniform* within the cohort). Additionally, the NAWM logarithmic normalization was better than no normalization and linear normalization at maintaining the consistency of both values and ranks within the cohort while a synthetic random variation of the acquisition parameter was applied (i.e., with a *heterogeneous* protocol within the cohort). Future PSR studies may consider reporting logarithmic normalized PSR (nPSR_ln_) values to potentially improve the comparability across studies.

## Introduction

The percentage of signal recovery (PSR) is a metric derived from dynamic susceptibility contrast (DSC) perfusion MRI that can be useful for differential diagnosis [1, 2, 3], molecular profiling [4], and treatment response assessment [5, 6] in brain tumors. However, the universalizability of PSR values is limited by their dependency on acquisition parameters [7, 8]. Normalizing the values of other MRI biomarkers, such as perfusion and diffusion metrics, using reference tissues is a well-established practice to limit the impact of acquisition heterogeneity [9]. In this study, we propose to normalize brain tumor PSR values using the PSR value of a reference tissue. We hypothesize that this will improve the consistency of PSR ranks and values across different protocols, and within cohorts with heterogenous protocols.

## Methods

### Patient cohort and image analysis

For this technical assessment, new analyses were performed on a previously reported cohort of 38 newly-diagnosed and recurrent contrast-enhancing gliomas [8]. Perfusion imaging was performed with dynamic spin-and-gradient echo-planar imaging (dynamic SAGE-EPI) and the two gradient echoes were used to generate multiple synthetic DSC datasets with desired synthetic acquisition parameters, as previously shown [8]. Synthetic DSC can compute DSC acquisitions with any desired combination of pulse sequence parameters and/or simulated preload administration [8]. Tumor segmentations including the contrast-enhancing tumor tissue were obtained using T_1_-subtraction maps [10], and then registered to the perfusion space.

Four reference tissue regions of interest (ROI’s) were evaluated: contralateral normal-appearing white matter (NAWM), cerebral falx, masticatory muscles, extracranial skin. The NAWM ROI (at least 2 cc) was drawn in the centrum semiovale according to a previously described “planar” method [9]. The cerebral falx ROI (at least 0.5 cc) included voxels appearing as contrast-enhancing and T_2_*-hypointense, not including vascular structures, drawn on two adjacent axial slices. The masticatory muscles ROI (at least 1 cc) was drawn on three adjacent slices bilaterally, excluding fat tissue, bone, and EPI susceptibility artifacts (e.g., areas of artifactual low T_2_* signal due to air-tissue interfaces). The extracranial skin ROI was obtained on a single slice, with a semi-automated method: the background voxels outside the skull were excluded via intensity thresholding, and the intracranial voxels were excluded by subtracting a brain mask obtained with automated skull stripping (*fsl bet*). All ROI’s were manually drawn by an expert neuroradiologist (reader 1, FS), using the pre-bolus baseline of dynamic SAGE-EPI and the post-contrast T_1_w registered to the perfusion space. The reader was blinded to the perfusion time-intensity curves to avoid any bias. Since PSR values bear a non-linear relation to some pulse sequence parameters [8], normalized PSR (nPSR) was generated not only with a linear (nPSR_linear_), but also with a logarithmic normalization (nPSR_ln_) that may adjust for this non-linear relationship when comparing protocols with heterogeneous pulse sequence parameters. nPSR_linear_ and nPSR_ln_ were calculated as follows:

$$\:{nPSR}_{linear}=\:\frac{{PSR}_{tumor}}{{PSR}_{reference}}\times\:100\%$$

$$\:{nPSR}_{ln}=\:\frac{\text{l}\text{n}\left({PSR}_{tumor}\right)}{\text{l}\text{n}\left({PSR}_{reference}\right)}\times\:100\%$$

### Statistical analyses

The ROI with the lowest PSR coefficient of variation (σ/µ) across patients was selected as the reference ROI of choice. The intra-reader agreement of the ROI of choice was calculated on the “standard” synthetic PSR-optimized protocol (FA 90° TE 30ms TR 1.5s P–) with a two-way mixed effects intraclass correlation coefficient for a single rater (ICC3) after the ROI drawing was repeated after a wash-out period of 3 months by reader 1 (FS). The inter-reader agreement with a reader 2 (JY) was calculated with a two-way random effects ICC for agreement (ICC2), as proposed in a previous study [9]. The agreement of PSR and nPSR values across different protocols (with a single reader) was calculated with ICC3, while the consistency of their ranks within the cohort was evaluated with a Spearman’s correlation coefficient (ρ) [8]. In other words, ICC3 measures whether two methods agree on the actual value of the measurement (e.g., whether a certain tumor exhibits PSR = 100% with both protocol A and protocol B), while ρ measures whether two methods agree on the ranks of the measurement (e.g., whether a certain tumor exhibits the highest PSR in the cohort with both protocol A and protocol B, regardless of changes in the actual value). First, the changes in values and ranks introduced by the normalization were quantified by computing ICC3 and ρ between tumor PSR and nPSR, using a single “standard” synthetic PSR-optimized protocol (FA 90° TE 30ms TR 1.5s P–) [8]. Second, PSR and nPSR were computed with different synthetic protocols, and the ICC3 and ρ were computed between the standard protocol and other protocols (with a *uniform* synthetic protocol for the whole cohort). This tested whether the normalization can improve the universalizability of PSR across cohorts acquired with different protocols. Third, ICC3 and ρ were calculated on PSR and nPSR obtained from bootstrapped cohorts while assigning to each subject a random acquisition parameter within a certain range (i.e., with *heterogeneous* synthetic protocols within each bootstrapped cohort). For each predetermined range of each acquisition parameter, the analysis was repeated for 1000 iterations to generate distributions of ICC3 and ρ to assess PSR inconsistencies within cohorts with heterogenous protocols.

## Results

### Selection of the reference tissue

**Fig. 1:**
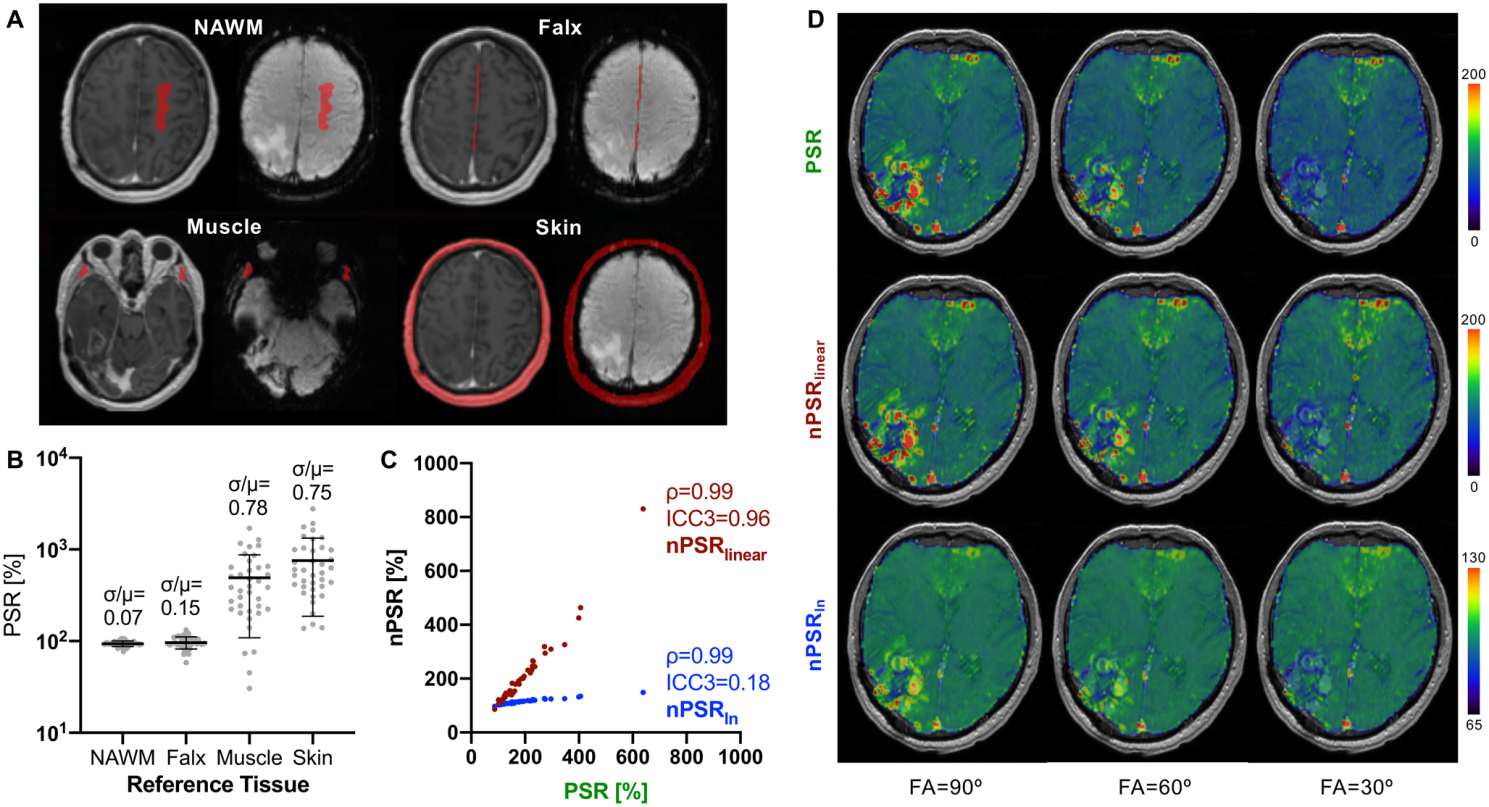
Preliminary analyses on normalization methods. Among the candidate reference tissues (**A**), the white matter (NAWM) showed the lowest variability across patients (**B**) and demonstrated to maintain the differences among tumors (**C**). A visual evaluation (**D** is a randomly-selected case) showed that a logarithmic normalization can mitigate the differences in PSR introduced by acquisition parameter variations (variations in FA, while the other parameters are maintained stable: TE 30 ms, TR 1.5 s, P–)

Median (interquartile range) size values of the reference ROI’s were as follows: NAWM 2.5 cc (2.2–3.9), Falx 1.0 cc (0.9–1.3), Muscle 2.8 cc (2.3–3.4), Skin 21 cc (18–24). Among the four candidate reference tissues (Fig. 1A), NAWM demonstrated the least inter-patient variability in PSR measurements when the experimental conditions were kept stable for all subjects (i.e., with a “standard” PSR-optimized synthetic protocol: FA 90°, TE 30 ms, TR 1.5 s, no preload simulation) [2, 8]. Since an ideal reference tissue aimed at correcting technical inconsistencies should have minimal inter-patient variability when the experimental conditions are uniform, NAWM was selected as reference tissue of choice for subsequent analyses (Fig. 1B). NAWM normalization showed a near-perfect inter-reader (nPSR_linear_: ICC2 0.995, nPSR_ln_: ICC2 0.997) and intra-reader (nPSR_linear_: ICC3 0.988, nPSR_ln_: ICC3 0.995) agreement (Table 1). The NAWM normalization, either linear (nPSR_linear_) or logarithmic (nPSR_ln_), did not introduce significant *rank* changes (ρ = 0.99, Fig. 1C) compared to PSR, using the default PSR-optimized protocol. However, nPSR *values* should not be compared to non-normalized values, since the agreement between PSR and nPSR is low, especially for the logarithmic normalization (ICC3 = 0.96 nPSR_linear_ and ICC3 = 0.18 nPSR_ln_, Fig. 1C). Visual evaluations of the normalized voxel-wise maps suggest that nPSR_ln_ can better mitigate the effect of protocol changes, compared to both nPSR_linear_ and PSR (Fig. 1D).

**Table 1:** Inter- and intra-reader agreement of normalized PSR measurements

Agreement type (Statistical test)	Linear normalization nPSR_linear_	Logarithmic normalization nPSR_ln_
Inter-reader agreement (ICC2)	0.995	0.997
Intra-reader agreement (ICC3)	0.988	0.995

### Usefulness of PSR normalization

A logarithmic PSR normalization using NAWM as a reference tissue was found to outperform no normalization and linear normalization in terms of value consistency and rank consistency in presence of protocol variations.

**Fig. 2:**
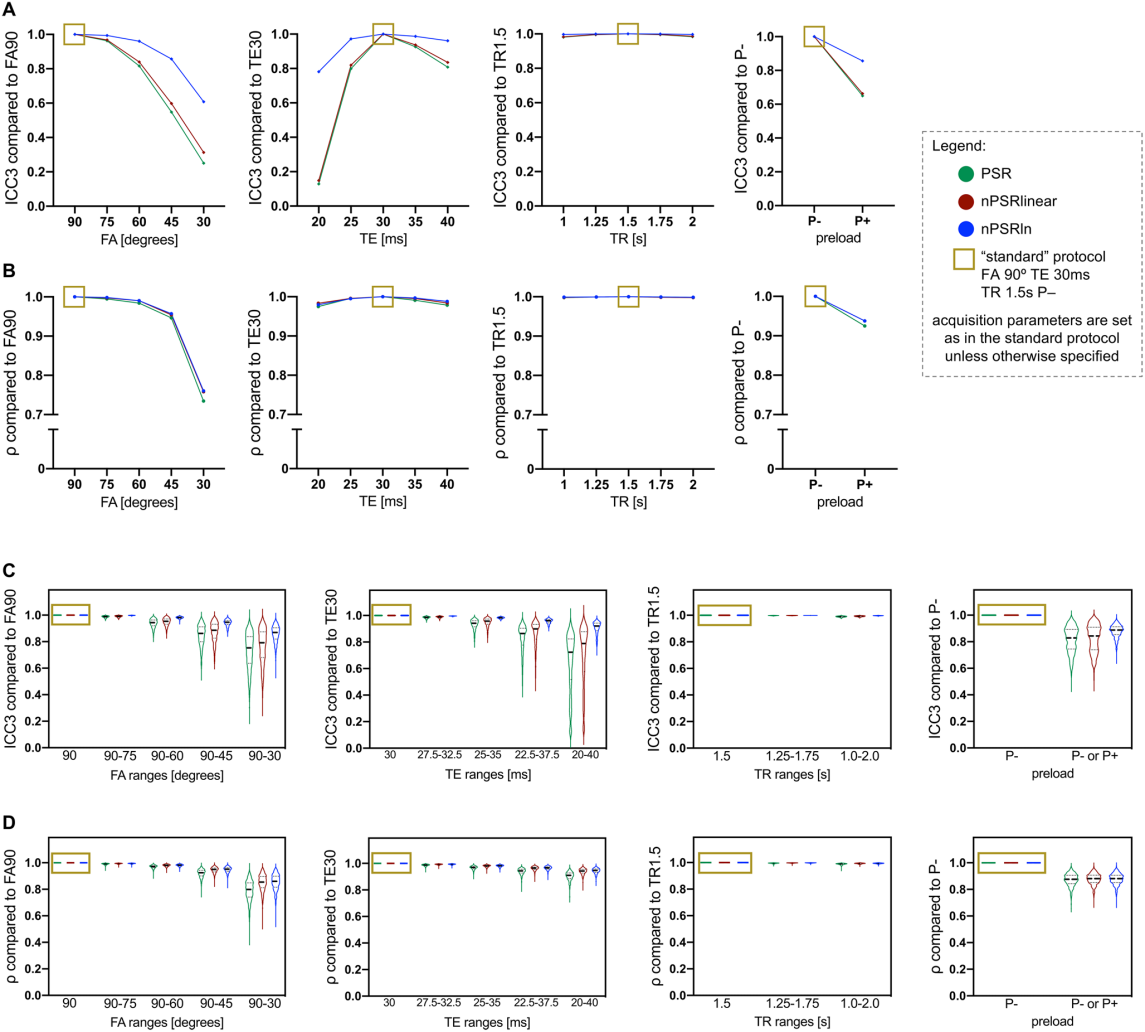
Assessment of white matter (NAWM) linear and logarithmic normalization. When uniformly varying acquisition parameters in the studied cohort, a logarithmic normalization (blue) improved the consistency of PSR values across protocols (**A**), while maintaining similar ranks (**B**), compared to the “standard” protocol (golden box). When varying acquisition protocols within each bootstrapped sample to obtain cohorts with heterogeneous protocols, a logarithmic normalization (blue violin plots) improved the consistency of both PSR values (**C**) and ranks (**D**) compared to the reference “standard” protocol (golden box)

When analyzing the cohort with different uniform protocols, nPSR_ln_ granted a better agreement of values obtained with different protocols: the blue line in Fig. 2A represents a higher ICC3 of nPSR_ln_ compared to nPSR (green) and nPSR_linear_ (red). The rank consistency was comparable among PSR, nPSR_linear_ and nPSR_ln_ (similar ρ in Fig. 2B) in this experiment. This means that the logarithmic normalization yields values that are more comparable across cohorts studied with different protocols and does not attenuate the differences among different tumors.

When analyzing bootstrapped cohorts with heterogeneous protocols, nPSR_ln_ achieved a better agreement among values calculated with varying ranges of acquisition parameters: the blue violin plots in Fig. 2C show distributions with higher ICC3 for nPSR_ln_ compared to nPSR (green violin plots) and nPSR_linear_ (red violin plots). Additionally, nPSR_ln_ also achieved a better rank consistency with heterogeneous protocols: the blue violin plots in Fig. 2D show distributions with higher ρ for nPSR_ln_ compared to nPSR (green violin plots) and nPSR_linear_ (red violin plots). This means that the logarithmic normalization better preserves the differences in PSR contrast when a certain cohort includes tumors studied with heterogeneous acquisition parameters.

## Discussion

This is the first report systematically testing several reference tissues and also the first one proposing a logarithmic normalization. NAWM logarithmic normalization should be preferred, as it mitigates the dependency of PSR values on acquisition protocols, while maintaining the differences and ranks among different tumors. Maintaining consistent values across protocols would benefit the universalizability of PSR values across different institutions adopting different acquisition protocols, allowing for a more consistent interpretation of such values in any given experimental condition. A linear normalization using NAWM as reference was used in prior studies [1, 2], although never validated for the purpose of across-protocol comparisons. Using NAWM as a PSR reference tissue may appear counterintuitive, since no contrast agent leakage happens in this tissue which can bear information about the post-bolus T_1_- to T_2_*- balance of the applied protocol. However, T_1_- and T_2_*- post-bolus effects are also seen in non-enhancing tissue, as a result of the leftover contrast agent *in the vasculature* [4], which can explain the usefulness of NAWM for PSR normalization. The cerebral falx might also be a reference tissue of interest to capture the information about the T_1_- to T_2_*- balance, given the peculiar post-bolus effects in the dura. However, the falx showed a higher inter-subject variability, probably linked to partial volumes effects due to the thinness of this anatomical structure. Importantly, the proposed method for NAWM ROI drawing showed excellent inter- and intra-reader agreement in the present study, and in a previous CBV study [9]. This study has some limitations: the design is retrospective, the sample size is relatively small, and the “synthetic” DSC methodology relies on some assumptions, as discussed in the original article [8]. Additionally, the study design tested the usefulness of NAWM normalization only as a strategy to mitigate technical inconsistencies, while it did not test whether it mitigates also potential non-tumoral biological heterogeneities (e.g., related to the cardiovascular system) across patients.

## Conclusions

A white matter normalization of PSR values, specifically with a logarithmic approach, may improve the consistency of both values and ranks across different DSC protocols. Future studies may report the values of both PSR and nPSR_ln_, to improve the universalizability of the reported values. Thresholds of nPSR_ln_ for clinical applications may be more reliably comparable across studies adopting different acquisition protocols and within cohorts imaged with heterogeneous protocols.

## Abbreviations

DSC: Dynamic Susceptibility Contrast
dynamic SAGE-EPI: Dynamic Spin-and-Gradient Echo-Planar Imaging
FA: Flip Angle
NAWM: Normal-Appearing White Matter
ICC3: Two-Way Mixed Single Score Intraclass Correlation Coefficient
nPSR: normalized PSR
nPSR_linear_: linear nPSR
nPSR_ln_: logarithmic nPSR
P: Preload
PSR: Percentage of Signal Recovery
ROI: Region of Interest
TE: Echo Time
TR: Repetition Time
ρ: Spearman’s correlation coefficient

## Declarations

### Editorial board members and editors

The authors have no editorial positions in this journal.

### Overlapping cohorts

Results from this cohort have been already reported and published, in articles that had a substantially different aim and methodology (Sanvito et al. Eur Radiol 2023, Sanvito et al. AJNR 2024).
